# Estrogen enhanced cell-cell signalling in breast cancer cells exposed to targeted irradiation

**DOI:** 10.1186/1471-2407-8-184

**Published:** 2008-06-30

**Authors:** Chunlin Shao, Melvyn Folkard, Kathryn D Held, Kevin M Prise

**Affiliations:** 1 Institute of Radiation Medicine, Fudan University, No.2094 Xie-Tu Road, Shanghai 200032, PR China; 2 Gray Cancer Institute, PO Box 100, Mount Vernon Hospital, Northwood, Middlesex, HA6 2JR, UK; 3 Dept Radiation Oncology, Cox 302, Massachusetts General Hospital, Harvard Medical School, 100 Fruit Street, Boston, MA 02114, USA; 4 Centre for Cancer Research and Cell Biology, Queen's University Belfast, 97 Lisburn Road, Belfast, BT9 7AB, UK

## Abstract

**Background:**

Radiation-induced bystander responses, where cells respond to their neighbours being irradiated are being extensively studied. Although evidence shows that bystander responses can be induced in many types of cells, it is not known whether there is a radiation-induced bystander effect in breast cancer cells, where the radiosensitivity may be dependent on the role of the cellular estrogen receptor (ER). This study investigated radiation-induced bystander responses in estrogen receptor-positive MCF-7 and estrogen receptor-negative MDA-MB-231 breast cancer cells.

**Methods:**

The influence of estrogen and anti-estrogen treatments on the bystander response was determined by individually irradiating a fraction of cells within the population with a precise number of helium-3 using a charged particle microbeam. Damage was scored as chromosomal damage measured as micronucleus formation.

**Results:**

A bystander response measured as increased yield of micronucleated cells was triggered in both MCF-7 and MDA-MB-231 cells. The contribution of the bystander response to total cell damage in MCF-7 cells was higher than that in MDA-MB-231 cells although the radiosensitivity of MDA-MB-231 was higher than MCF-7. Treatment of cells with 17β-estradiol (E2) increased the radiosensitivity and the bystander response in MCF-7 cells, and the effect was diminished by anti-estrogen tamoxifen (TAM). E2 also increased the level of intracellular reactive oxygen species (ROS) in MCF-7 cells in the absence of radiation. In contrast, E2 and TAM had no influence on the bystander response and ROS levels in MDA-MB-231 cells. Moreover, the treatment of MCF-7 cells with antioxidants eliminated both the E2-induced ROS increase and E2-enhanced bystander response triggered by the microbeam irradiation, which indicates that ROS are involved in the E2-enhanced bystander micronuclei formation after microbeam irradiation.

**Conclusion:**

The observation of bystander responses in breast tumour cells may offer new potential targets for radiation-based therapies in the treatment of breast cancer.

## Background

The radiation-induced bystander effect is the appearance of a biological response in nonirradiated cells neighbouring irradiated cells [1]. The response has been demonstrated in cultured cells and tissues by using different irradiation approaches including low fluences of α-particles [2,3], γ-rays [4,5], heavy ions [6,7], and targeted microbeams which allow cells to be individually irradiated through either the nucleus or cytoplasm [8-10]. Many endpoints have been reported for the bystander responses, including DNA damage markers [11,12] cell death [13], increases in sister chromatid exchanges [14,15], micronuclei [11,16,17], mutations [18,19] genomic instability [20,21], malignant transformation [22,23] and gene expression [24].

Recently, we have found that irradiation through the cytoplasm of a cell has a similar probability of triggering a bystander response to that when the nucleus is directly irradiated [10,25]. However, the mechanisms underpinning the bystander effect are still unclear, although cell-to-cell communication [26-28] and several signaling factors such as cytokines [29], reactive oxygen species (ROS) [30,31] and nitric oxide (NO) [32,33] have been identified as playing roles. These findings may be of particular importance for exposures at environmentally relevant low doses where cells at risk are traversed by only single tracks of radiation at any one time [34]. Bystander responses may also be relevant to the therapeutic use of radiation because a mechanistic understanding of the effects may lead to approaches to enhance bystander responses in tumours and also possibly to protect surrounding normal tissues.

Although evidence shows that bystander responses can be induced in many types of cells, it is not known whether there is a radiation-induced bystander effect in breast cancer cells, where the radiosensitivity may be dependent on the role of the cellular estrogen receptor (ER)[35]. Estrogens and anti-estrogens are important components of breast cancer development and treatment. The experimental data are contradictory as to whether estrogens and anti-estrogens alter the radiation response of breast cancer cells. It has been reported that 17β-estradiol (E2) prevents radiation-induced apoptosis of ER-positive MCF-7 breast cancer cells, probably mediated through the plasma membrane ER [36]. However, a number of studies have indicated that estradiol treatment increases the radiosensitivity of MCF-7 cells [37-39]. Conversely, treatment with the anti-estrogen, tamoxifen reduces or does not alter the radiosensitivity of MCF-7 cells [37,40], although it has no effect on radiosensitivity of ER-negative MDA-MB-231 breast cancer cells [41]. However, none of these studies have considered the response of breast cells and their modulation by estradiols and anti-estradiols after low dose radiation exposure.

In the present work, we used a charged particle microbeam to deliver exact numbers of helium ions through the nuclei of restricted numbers of MCF-7 and MDA-MB-231 breast cancer cells. We found that radiation-induced bystander responses were generated in both cell lines and that treatment with E2 and/or tamoxifen influenced the bystander response through a ROS-mediated pathway in only MCF-7 cells but not in MDA-MB-231 cells.

## Methods

### Cell culture and treatments

ER-positive MCF-7 and ER-negative MDA-MB-231 breast cancer cells (obtained from Cancer Research UK) were cultured in DMEM medium supplemented with 10% (v/v) foetal calf serum (FCS), 2 mM L-glutamine, 100 units/ml penicillin, and 100 μg/ml of streptomycin. Cells were grown in a humidified atmosphere with 5% CO_2 _in air at 37°C. One day prior to microbeam irradiation, plateau phase cells were seeded at a low density in a φ5 mm central area of the specially designed microbeam dish consisting of a 3 μm thick Mylar film base [42]. The region prepared for cell seeding had been pre-treated with 1.7 μg/cm^2 ^Cell-Tak adhesive (Collaborative Biomedical Products, Bedford, MA, USA). One hour after cell seeding, 2 ml of medium was added into the microbeam dish. In some experiments, cells were treated 24 h before and after microbeam irradiation with 10 nM 17-β estradiol (E2), 50 nM tamoxifen (TAM), 150 U/ml of the antioxidants superoxide dismutase (SOD) and catalase (CAT), or the combination of E2 with either TAM or antioxidants (all from Sigma, Poole, Dorset, UK). The concentration of E2 utilized for this study was within the range of physiological concentrations which could saturate the ER. TAM with a concentration of 5-fold excess was used to displace E2 from its receptor. These concentrations were also widely applied in other studies [37,43].

### Microbeam irradiation

The Gray Cancer Institute microbeam system was used for this study and details of the experimental set-up have been described elsewhere [42,44]. To enable individual nuclei to be identified by the microbeam imaging system, the fully-attached cells were stained with 0.2 μg/ml Hoechst 33342 for 1 hr prior to irradiation. Excess stain was removed by washing the cells with serum free medium containing 10 mM HEPES before irradiation, and cells were maintained in this medium during microbeam irradiation. Typically, 1200 ± 50 (mean ± SE) individual cells in total were scanned in the microbeam dish just before irradiation. A fraction of cells, from 1% to 100% of the cells in the population, were individually irradiated through the center of the nucleus with a precise number of helium-3 ions (^3^He^2+^) with an LET of 100 keV/μm. Using these ions, 99% of cell nuclei could be precisely targeted with an accuracy of ± 2 μm. Immediately after irradiation, the culture medium was replaced with 2 ml of complete medium with or without drugs and incubation continued until treatment for micronucleus analysis.

### Micronucleus assay

The cytokinesis block technique was used to assay for micronuclei (MN) *in situ*. 24 h after irradiation, the culture medium in the microbeam dishes was replaced with medium containing 1 μg/ml cytochalasin-B. The cells were incubated for a further 48 h then fixed with methanol: acetic acid (9:1 (v/v)) for 20 min. After air-drying, cells were stained with 10 μg/ml acridine orange plus 10 μg/ml Hoechst 33342 for 5 min. This Hoechst treatment enhanced visualisation of both nucleus and micronucleus. MN were scored in binucleated (BN) cells and classified according to the criteria of [45]. The MN yield, Y_MN_, was calculated as the ratio of the number of MN to the number of scored BN cells.

### ROS assay

To investigate the possible role of ROS in the E2-induced effect, 2 × 10^5^MCF-7 or MDA-MB-231 cells were treated with 10 nM E2 or E2 plus 150 U/ml SOD and CAT for 24 h. Then, the intracellular ROS level in the cells was measured *in situ *by using 5',6'-chloromethyl-2',7'-dichlorodi-hydro-fluorescein diacetate (CM-H_2_DCFDA, Molecular Probes Inc.). Briefly, cells were treated with 3.5 μM CM-H_2_DCFDA for 20 min at 37°C and then washed with FCS free medium for 15 min. The fluorescence images of at least 100 randomly selected cells per dish were captured using a 3 CCD cooled colour camera (Photonic Science Ltd, East Sussex, UK) attached to a fluorescence microscope (Zeiss Axioskop). The exposure conditions were standardised to allow quantitative comparisons of the relative fluorescence intensity of the cells between groups.

### Statistical analysis

Statistical analysis was done on the means of the data obtained from at least three independent experiments. Two replicates were counted for each experimental point in each experiment to determine the micronucleus yield. All results are presented as means ± SE. Significance was assessed using the Student's t-test at *P *< 0.05.

## Results

### Radiation induced bystander effect

**Figure 1:**
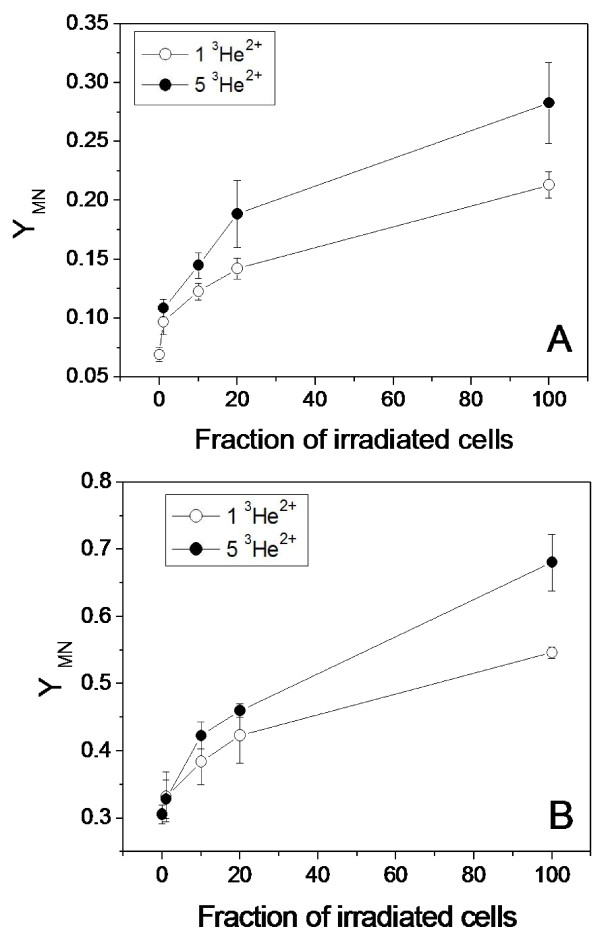
**Radiation-induced MN formation in breast cancer cells**. Different fractions of cells within the populations of MCF-7 **(A) **and MDA-MB-231 **(B) **were individually irradiated with 1 or 5 ^3^He^2+ ^particles, respectively.

When a fraction of either MCF-7 or MDA-MB-231 cells within a population were individually targeted through their nuclei with a precise number of helium particles, MN were induced. The yields of MN in the targeted populations increased non-linearly with the fraction of irradiated cells (Fig. 1). When 100% of cells were irradiated with 1 or 5 ^3^He^2+ ^particles, the yield of radiation-induced MN in the MDA-MB-231 cells was higher than that in the MCF-7 cells, indicating that the MDA-MB-231 cells have a higher radiosensitivity than MCF-7 cells. In contrast, it was calculated from the data in Fig. 1 that, when 12 cells (1% of the population) were individually targeted with 1 He^2+ ^particle, although not every targeted cell leads to the production of a MN, MN were still observed in an additional 33 MCF-7 cells or 29 MDA-MB-231 above background which provides clear evidence that MN are produced in non-targeted bystander cells at a similar level in both cell lines.

If no bystander effect occurred in the population where 100% of cells were targeted individually, the yields of MN when only a fraction of the cells were irradiated can be mathematically predicted. This uses the MN yield when 100% of cells were irradiated by the method described previously [32] and the yields are listed in Table 1. In comparison to the data in Fig. 1, the predicted MN yields are smaller than the measured values for both the MCF-7 and MDA-MB-231 cell lines. The difference in yield of MN between the measured value and the predicted value is defined as the yield of bystander MN (see Table 1). In general, the bystander MN yield increased with the fraction of irradiated cells and with the radiation dose (number of particles delivered to the targeted cells). In addition, although the bystander MN yield in MBA-MD-231 cells was higher than that of MCF-7 cells, the bystander MN as a percentage of the total MN in the MDA-MB-231 population was less than that of MCF-7 cells. For example, when 1 to 20% of cells in a population are irradiated with 1 or 5 ^3^He^2+ ^particles, the bystander MN as a percentage of the total MN ranges from 5.8% to 18.9% for the MDA-MB-231 cells, and from 27.1% to 40.1% for the MCF-7 cells. Therefore, MCF-7 cells are more effective in generating a bystander response than MDA-MB-231 cells, which is in contrast to the differences in radiosensitivity between the two cell lines.

**Table 1:** The predicted yield of MN and the measured yield of bystander MN.

	MCF-7 cells				MDA-MB-231 cells			
	Predicted Y_MN_		Bystander Y_MN_		Predicted Y_MN_		Bystander Y_MN_	

Fraction of irradiated cells	1 ^3^He^2+^	5 ^3^He^2+^	1 ^3^He^2+^	5 ^3^He^2+^	1 ^3^He^2+^	5 ^3^He^2+^	1 ^3^He^2+^	5 ^3^He^2+^

1%	0.071	0.071	0.026 (27.1%)	0.037 (34.3%)	0.308	0.309	0.024 (7.2%)	0.019 (5.8%)
10%	0.084	0.091	0.039 (31.9%)	0.054 (37.5%)	0.330	0.343	0.054 (14.0%)	0.080 (18.9%)
20%	0.098	0.11	0.044 (31.0%)	0.077 (40.1%)	0.354	0.380	0.069 (16.4%)	0.080 (17.4%)

A fraction of cells within the MCF-7 and MDA-MB-231 populations were individually irradiated with a precise number of ^3^He^2+ ^particles. Data in parentheses are the bystander MN as a percentage of total MN.

### Influence of E2 and TAM on the bystander response

**Figure 2:**
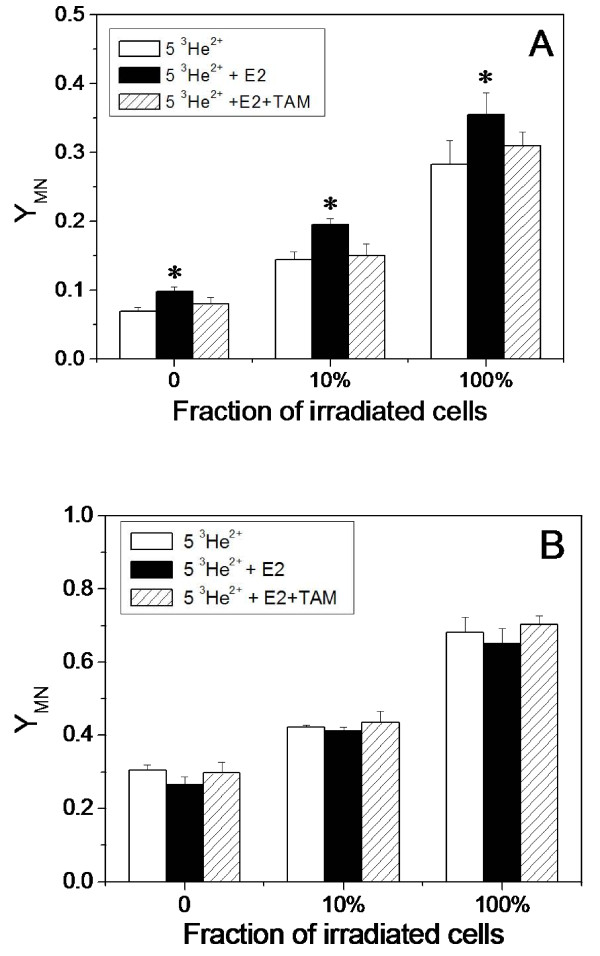
**Influence of E2 and TAM on radiation-induced MN formation**. A fraction of MCF-7 (**A**) or MDA-MB-231 (**B**) cells were individually irradiated with 5 ^3^He^2+ ^particles without or with pre-treatment with E2 or E2+TAM (*, *P *< 0.05 compared to the MN yield without E2 treatment).

E2 is an important factor involved in breast cancer development. To investigate whether E2 can influence the radiation-induced bystander effect, we treated cells with E2 for 24 h before irradiation and afterwards until assayed for MN. Results are illustrated in Fig. 2 where a fraction of either MCF-7 or MDA-MB-231 cells were individually irradiated with 5 ^3^He^2+ ^particles. The E2 treatment itself significantly increased the MN background in MCF-7 cells (*P *< 0.05) but not in MDA-MB-231 cells, which is in agreement with a previous report that E2 increases the formation of MN in ER-positive MCF-7 cells [46]. When 100% of the cells were irradiated, E2 also increased the production of MN in MCF-7 cells and this increase is larger than the E2-enhanced MN background (Fig. 2A). However, E2 had no influence on MDA-MB-231 cells (Fig. 2B). Therefore, E2 increases the radiosensitivity of MCF-7 cells but not MDA-MB-231 cells.

Interestingly, the MN yield was also increased by E2 treatment when 1% or 10% of cells in the MCF-7 population were irradiated. According to the MN yield when 100% of cells were irradiated in the presence of E2, the predicted yield of MN, assuming no bystander effect occurred in the fraction-irradiated population, and the yield of bystander MN in the E2-treated MCF-7 cells were calculated and are listed in Table 2.

**Table 2:** The predicted yield of MN and the measured yield of bystander MN in MCF-7 cells.

Fraction of irradiated cells	Predicted Y_MN_		Bystander Y_MN_	
	
	1 ^3^He^2+^	5 ^3^He^2+^	1 ^3^He^2+^	5 ^3^He^2+^
1%	0.010	0.010	0.035 (25.8%)	0.057 (36.2%)
10%	0.114	0.124	0.067 (37.1%)	0.072 (36.7%)

A fraction of cells within the population, which had been treated with E2 for 24 h, were individually irradiated with a precise number of ^3^He^2+ ^particles. Data in parentheses are the bystander MN as a percentage of total MN.

Comparing the data in table 2 with those in table 1, it is seen that treatment of MCF-7 cells with E2 increased the yield of bystander MN when 1% or 10% of the cells were individually irradiated with 1 or 5 ^3^He^2+ ^particles. For example, when 1% of cells were irradiated with 1 ^3^He^2+ ^particle, E2 increased the bystander MN yield from 0.0262 to 0.0347. This E2-enhanced bystander response could result from the E2-enhanced radiosensitivity of MCF-7 cells.

Figure 2 also illustrates that when the cells were treated with TAM to compete for ER with E2, both radiosensitivity and the radiation-induced bystander effect on MCF-7 cells were diminished to the levels in the absence of E2 treatment. In addition, treatment of cells with TAM plus E2 did not show any influence on radiation damage to MDA-MB-231 cells. Treatment with TAM alone had no influence on the radiosensitivity of either cell line (data not shown).

### ROS contributes to the E2-enhanced radiation effect in MCF-7 cells

**Figure 3:**
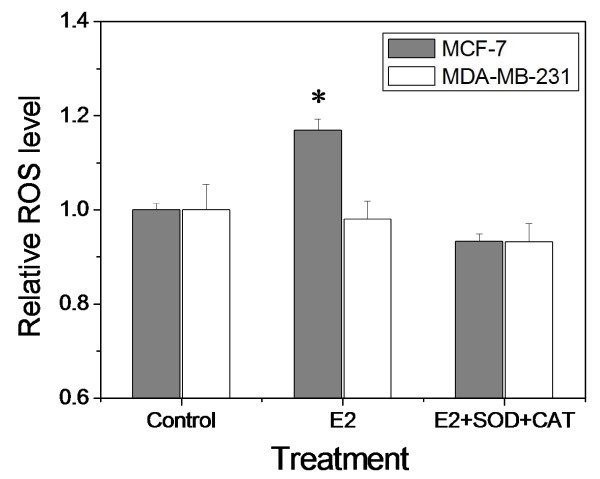
**Relative ROS levels**. MCF-7 cells and MDA-MB-231 cells were treated with E2 or the mixture of E2 and SOD plus CAT (*, *P *< 0.01 compared to the control without E2 treatment).

To investigate the signaling factors involved in the E2-enhanced cellular damage in MCF-7 cells, we measured the levels of intracellular ROS in cells with or without E2 treatment. Fig. 3 illustrates that, when MCF-7 cells were treated with 10 nM E2 for 24 h, the levels of ROS increased 1.17 fold relative to controls. When SOD and CAT were present during E2 treatment, the level of ROS in MCF-7 cells was decreased to 93% of the control. In the ER-negative MDA-MB-231 cells, E2 treatment had no influence on the levels of ROS although treatment with antioxidants decreased the intracellular levels of ROS (not significant).

**Figure 4:**
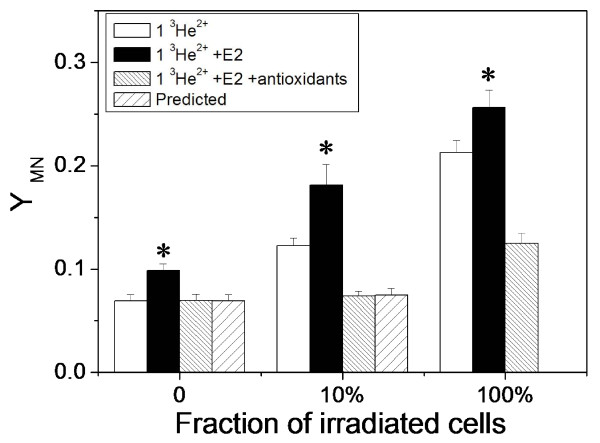
**Influence of E2 and antioxidants on formation of radiation-induced MN in MCF-7 cells**. A fraction of cells were individually irradiated with 1 ^3^He^2+ ^particle. The predicted yields of MN assuming no bystander effect were calculated from the yield of MN when 100% of cells were irradiated with antioxidant treatment (*, *P *< 0.05 compared to the MN yield of MCF-7 cells without E2 treatment).

To investigate whether the E2-induced ROS contributes to the radiation-induced bystander response, we measured the yield of MN in the fraction-irradiated MCF-7 population treated with SOD and CAT antioxidants in the presence of E2. Results are shown in Fig. 4 where 10% and 100% of MCF-7 cells were individually irradiated with 1 ^3^He^2+ ^particle. The antioxidant treatment eliminated the E2-enhanced MN background, which corresponds to the result in Fig. 3 and hence confirms that ROS are the source of E2-induced MN in MCF-7 cells. Importantly, when 100% of MCF-7 cells were irradiated, the antioxidant treatment not only diminished E2-enhanced MN formation but reduced the MN to a very low level, which indicates that ROS contribute to the DNA damage induced by direct radiation. From the measured MN yields when 100% of the cells were irradiated with antioxidant treatment, we calculated the predicted MN yield assuming no bystander effect occurred when 10% of cells were irradiated and found that this matched the actual MN yield (Fig. 4). Accordingly, ROS not only contribute to the direct radiation-induced cellular damage but are also involved in the E2-enhanced bystander response of MN induction in MCF-7 cells.

## Discussion

This study finds that, when a fraction of cells are precisely irradiated with a counted number of ^3^He^2+ ^particles, the bystander response, measured as an increased yield of MN, can be produced in both non-irradiated MCF-7 and MDA-MB-231 cells, independent of ER status. Radiation-induced signalling factors could contribute to these bystander responses. For example, it has been reported that the conditioned medium from irradiated MCF-7 or MDA-MB-231 cells contained twofold more TGF-β1 than that from nonirradiated cells [47]. Also significant evidence for the role of paracrine signalling in response to radiation exposure has been reported (See [48]for a review). Our previous studies have shown that TGF-β1 is an important t bystander signaling factor which can further raise the level of intracellular ROS and NO and then cause DNA damage [49,50].

Interestingly, the contribution of the bystander response to cellular damage in ER-positive MCF-7 cells was higher than that in ER-negative MDA-MB-231 cells. Moreover, treatment of cells with E2 increases the radiation-induced bystander damage in the ER-positive MCF-7 cells but not in the ER-negative MDA-MB-231 cells, and this E2-enhanced bystander response in MCF-7 cells can be reversed by tamoxifen, an anti-estrogen reagent.

The effects of E2 on breast cancer cells are complicated and sometimes inconsistent. It has been reported that E2 acts as both a growth factor and a survival factor for breast cancer cells and prevents cell death from apoptosis [36]. However, E2 is a carcinogen and can induce multiple forms of DNA damage such as single strand breaks, chromosome aberrations, and gene mutations [51]. We find here that the effect of E2 on DNA damage is dependent on the ER status of the cells. E2 can induce MN in ER-positive MCF-7 cells but not in ER-negative MDA-MB-231 cells, which is in agreement with other studies [46,52]. Fisher *et al. *suggested that the E2-induced formation of MN was not due to the chromosome damaging activity of estradiol but to stimulation of MCF-7 proliferation. For our experiment, the cells were seeded on dishes pre-treated with Cell-Tak so that the cell plating efficiency was ~100%; shortly after irradiation, the cell growth was blocked by cytochalasin-B. Under these conditions, we did not find that the cell proliferation was increased by E2. Thus, E2-induced MN in MCF-7 cells reported here could not result from increased cell proliferation but is generated from the cytotoxic effect of E2.

Direct evidence in the present study shows that E2-induced responses of ROS and MN induction are ER-dependent. Also, the ROS produced contribute to E2-induced DNA damage in MCF-7 cells since this damage was reduced by antioxidants. It has been reported that several types of free radical-mediated DNA damage can be induced by estrogens and their metabolites. For instance, 8-hydroxyguanine, formed by hydroxyl radical reaction with guanine bases, is increased over control values in DNA of cells incubated with estradiol [53]. In fact, the genotoxic activity of E2 is thought to be tightly linked to an oxidative element probably involving an ER-mediated mechanism. At physiological concentrations of E2, an ER-mediated pathway is more capable of inducing increases in ROS through the regulation of antioxidant genes [43]. E2 can be preferentially oxidised by cytochrome P-450 to produce 2-hydroxyestradiol that reacts with DNA and can be further oxidized to quinone acocmpanied by generation of ROS [54].

Moreover, we find that the E2-induced ROS contribute to the E2-enhanced radiosensitivity and bystander response of MCF-7 cells (Fig. 4). However, ROS may not be the only signalling factor involved in the bystander response because they can be produced in both irradiated and non-irradiated cells after E2-treatment. We have recently found that nitric oxide (NO) can be produced in irradiated MCF-7 cells. Even when only 1% of cells are targeted, the NO level in the whole cell population can be increased by 19% relative to controls (data not shown). One possibility is that ROS increases not only the E2-enhanced radiosensitivity of MCF-7 cells but also the cellular sensitivity to other toxic factors including NO and its downstream products induced by radiation so that the bystander response of cellular damage can be increased by the E2-treatment. As E2 is an essential factor for the development and growth of breast cancer cells, our finding that E2 increases the radiation-induced bystander effect may have relevance for the future development of radiation-dependent therapies for breast cancer.

Bystander responses have also been observed in breast tumour cells after various chemical treatments. For example, using a co-culture approach, Chhipa and Bhat reported increased bystander cell killing in MCF-7 cells co-culutred with MDA-MB-231 cells which had been pretreated with 5-fluorouracil (5-FU) and this was mediated by the Fas/FasL system [55]. In another study pre-treatment of breast cells with paclitaxel, leads to increased ROS levels and this could produce increased cell killing in non paclitaxel treated bystander cells [56].

## Conclusion

We report here evidence for radiation-induced bystander responses in breast tumour cells. In MCF-7 cells, the bystander response could be enhanced by treatment with E2 and quenched by the addition of TAM in an ROS dependent manner, neither of which modulated the bystander response in MDA-MD-231 cells. The observation of bystander responses in breast tumour cells may offer new potential targets for radiation-based therapies in the future.

## Abbreviations

ROS: reactive oxygen species; E2: 17β-estradiol; ER:  Estrogen receptor; TAM: Tamoxifen, NO: nitric oxide; MN: micronucleus; BN: binucleate.

## Competing interests

The authors declare that they have no competing interests.

## Authors' contributions

CS performed experiments, MF designed microbeam study, KDH and KMP designed study, analysed data. CS wrote the manuscript and KDH and KMP revised manuscript.

## Pre-publication history

The pre-publication history for this paper can be accessed here:


